# Evaluating the Quality of Artificial Intelligence-Generated Information on Cleft Lip and Palate: A Comparative Cross-Sectional Study

**DOI:** 10.3390/healthcare14111535

**Published:** 2026-06-01

**Authors:** Amir Bilder, Michal Almos, Ahmad Hija, Andrei Krasovsky, Nidal Zeineh, Tal Capucha, Omri Emodi

**Affiliations:** 1 Department of Oral and Maxillofacial Surgery, Rambam Health Care Campus, Haifa 3109601, Israel; a_bilder@rmc.gov.il (A.B.); m_even@rmc.gov.il (M.A.); a_krasovsky@rmc.gov.il (A.K.); n_zeineh@rmc.gov.il (N.Z.); t_capusha@rmc.gov.il (T.C.); o_emodi@rmc.gov.il (O.E.); 2 The Ruth & Bruce Rappaport Faculty of Medicine, Technion—Israel Institute of Technology, Haifa 3109601, Israel

**Keywords:** cleft lip and palate, large language model, ChatGPT, GPT-4o, Gemini, health information quality, Global Quality Score, inter-rater reliability

## Abstract

Background/Objectives: Large language models (LLMs) are increasingly consulted for information about cleft lip and palate (CLP), yet the reliability of their outputs across clinical domains has not been evaluated. This study aimed to compare the quality of CLP-related information generated by GPT-4o and Gemini 2.5 Pro across multiple thematic domains using a validated quality instrument and a reliability-first analytic framework. Methods: Fifty-four standardized CLP questions across six domains were submitted to GPT-4o (OpenAI) and Gemini 2.5 Pro (Google DeepMind) on 25 September 2024 via their public interfaces, using new, history-free sessions and default settings, yielding 108 responses. Three independent, CLP-experienced raters scored each response using the Global Quality Score (GQS; 1–5 scale assessing accuracy, completeness, and clinical usefulness). Before comparing models, we applied a reliability-first filter: only domains where all three raters showed substantial agreement (Fleiss’ kappa [κ] ≥ 0.60) were included in statistical comparisons. Domains that failed this threshold were analyzed qualitatively to identify the source of disagreement. A descriptive taxonomy of errors was developed for low-scoring responses. Results: Three domains met the reliability threshold (General Care Information, General Cleft Information, and Pre-Treatment Information; 30 paired questions). Both models performed at a high and practically equivalent level: GPT-4o median GQS 4.33 (IQR 4.00–5.00) versus Gemini 2.5 Pro 5.00 (IQR 4.00–5.00); the difference was not statistically significant (Wilcoxon V = 139.00, *p* = 0.691; Hodges–Lehmann median difference 0.00, 95% CI −0.33 to 0.67). Three domains were excluded because rater agreement was insufficient; qualitative review showed this reflected genuine clinical practice variation rather than clear model errors. The most common inaccuracies were overgeneralization of outcomes, outdated surgical timing, and omission of multidisciplinary team roles. Conclusions: Both models provided high-quality CLP information in domains supported by clinical consensus, indicating they may serve as useful adjuncts for general patient and family counseling. Clinicians should, however, verify any treatment-specific content against current institutional protocols before relaying it to patients. Future research should assess readability, alignment with health literacy, and patient comprehension of AI-generated CLP information.

## 1. Introduction

Large language models (LLMs) can generate natural-language responses to complex prompts and are increasingly used by both patients and clinicians as a primary source of health information [1,2]. These models, including GPT (OpenAI) and Gemini (Google DeepMind), have demonstrated the ability to produce fluent, contextually relevant medical text that can be difficult to distinguish from expert-authored content [3,4]. In the fields of oral and maxillofacial surgery and related disciplines, initial studies have demonstrated moderate-to-high performance on select, protocol-driven topics [5,6,7]. However, issues such as hallucinated content, citation fabrication, and factual inaccuracies persist, and the performance of these models varies significantly across different specialties and clinical contexts [8,9].

The evaluation of AI-generated health information has gained substantial research attention. Several validated instruments exist for this purpose, including the DISCERN tool for treatment-choice information [10], the JAMA benchmarks for website quality [11], and the Global Quality Score (GQS), a 5-point scale assessing overall accuracy, completeness, clarity, and clinical usefulness [12,13]. The GQS has been widely applied in recent studies evaluating LLM outputs in otolaryngology [6], oral surgery [14], medication-related osteonecrosis [15], and post-surgical follow-up [16]. However, the reliability of expert ratings using these instruments is often assumed rather than formally tested, and studies rarely report or act upon inter-rater agreement metrics.

Cleft lip and palate (CLP) care is a multidisciplinary, longitudinal pathway that spans infancy through adulthood and often involves heterogeneous protocols across centers and regions [17,18,19,20]. Information-seeking by families and patients commonly includes general disease concepts, perioperative preparation, postoperative care, and long-term expectations. Because some aspects of CLP management are supported by strong consensus (e.g., primary lip repair timing, team composition) while others remain practice-variable or evidence-limited (e.g., secondary alveolar bone grafting timing, speech surgery indications) [21,22], CLP provides a particularly stringent test case for evaluating when LLM-generated information is dependable for patient-facing use.

Despite the growing literature on LLMs in healthcare, no study has systematically evaluated AI-generated CLP information across multiple clinical domains while incorporating formal inter-rater reliability assessment as a prerequisite for valid comparison. Moreover, prior studies have generally reported aggregate quality scores without characterizing the types of errors that LLMs produce—information that is arguably more clinically actionable than summary statistics alone. The potential consequences of inaccurate AI-generated health information extend beyond inconvenience, particularly for the vulnerable population of parents and caregivers of children with CLP. Families receiving a new diagnosis often turn to online sources and conversational AI tools as a first step, before specialist appointments can be arranged [20]. In this context, overly optimistic prognoses may generate false expectations about surgical outcomes, outdated guidance on feeding or surgical timing may delay appropriate intervention, and incomplete descriptions of the multidisciplinary team may result in families failing to access essential services such as audiology or psychology. The psychological burden of navigating a complex, multi-year treatment pathway is well documented [18], and misinformation during critical decision-making windows—such as the neonatal period or pre-operative preparation—may compound distress and undermine informed consent. For clinicians, understanding which domains of LLM output are reliably accurate and which carry an elevated risk of error is therefore of direct practical importance, as they are increasingly called upon to contextualize or correct AI-generated content that patients have already encountered [23,24].

The objective of this study was to assess and compare the quality of CLP-related information generated by GPT-4o and Gemini 2.5 Pro across multiple information domains, using a validated quality instrument and a reliability-first analytic approach. We hypothesized that overall information quality would not differ between models in domains where inter-rater agreement supported valid aggregation of ratings. A secondary objective was to characterize the types of informational errors produced by both models to inform clinical guidance on LLM use in CLP counseling.

## 2. Materials and Methods

### 2.1. Study Design and Question Development

This cross-sectional comparative study evaluated the quality of AI-generated responses to standardized CLP-related questions. A set of 54 questions was developed by a panel of CLP-experienced clinicians to reflect common information needs of patients and practitioners across six thematic domains: (1) General Cleft Information (10 questions), (2) Pre-Treatment Information (10 questions), (3) During-Treatment Information (10 questions), (4) Post-Treatment Information (10 questions), (5) General Care Information (10 questions), and (6) Case Scenarios requiring integration across domains (4 scenarios). Questions were drafted to mirror language commonly used by patients and caregivers and were reviewed for clinical relevance and clarity by all investigators prior to finalization. To minimize potential question formulation bias, the panel followed a structured approach: each question was independently drafted by two investigators (A.B. and M.A.) without reference to each other’s versions, after which both versions were reconciled through group discussion. Questions were framed in neutral, non-leading language and were piloted on a convenience sample of five junior medical students to verify lay-audience comprehensibility before finalization. No external validation panel or patient-advisory input was sought; this is acknowledged as a limitation. The complete list of 54 questions is provided in Appendix A; a representative subset of questions with corresponding AI-generated responses is provided in Supplementary Materials S1.

### 2.2. LLM Response Generation and Blinding

All 54 questions were submitted to two commercially available LLMs on 25 September 2024: GPT-4o (OpenAI, San Francisco, CA, USA; accessed via ChatGPT at chat.openai.com, accessed on 26 September 2025) and Gemini 2.5 Pro (Google DeepMind, Mountain View, CA, USA; accessed via gemini.google.com). To support reproducibility, the following procedural parameters are reported in full: (i) GPT-4o was accessed using a paid ChatGPT Plus subscription (GPT-4o model, version current as of 25 September 2024); Gemini 2.5 Pro was accessed using a standard Google account with no Gemini Advanced subscription. (ii) Both models were accessed from Israel using the English-language interface; no region-specific filtering or language localization was applied. (iii) Chat history and memory features were explicitly disabled prior to each session in both platforms, so that prior interactions could not influence responses. (iv) No browsing, code-execution, or third-party plugins were enabled. (v) Each question was submitted as a standalone prompt in a newly initiated conversation session, with no system prompt, preamble, or introductory framing beyond the question text itself. (vi) No responses were regenerated; the first output produced for each question was recorded verbatim. (vii) In the small number of instances (*n* = 3 across both models) where the model declined to answer or produced an incomplete response on first submission, the question was re-submitted once in a new session; all such cases are flagged in Supplementary Materials S1. Responses were exported verbatim, stripped of model identifiers, assigned unique alphanumeric codes, and randomized prior to scoring. Because the study analyzed outputs from publicly available software and involved no human participants or patient records, institutional review board approval was not required, Sample questions and representative AI responses are provided in Supplementary Materials S1; individual rater scores are available in Supplementary Materials S2.

### 2.3. Outcome Measure and Rating Procedure

The primary outcome was the Global Quality Score (GQS), a 5-point ordinal scale validated for assessing the overall quality of health information, including accuracy, completeness, clarity, and clinical usefulness (1 = very poor, generally poor quality and very unlikely to be of benefit to patients; 2 = poor, generally poor quality but some information present; 3 = moderate, suboptimal flow but some important information adequately discussed; 4 = good, good quality and flow, most important topics covered, with some inaccuracies; 5 = excellent, accurate and complete information with excellent flow) [12,13]. Three independent raters with clinical expertise in CLP management scored all 108 responses while blinded to model identity and to each other’s ratings. The three raters were a senior oral and maxillofacial surgeon with over ten years of dedicated CLP experience, a speech-language pathologist specializing in velopharyngeal dysfunction and post-palatoplasty outcomes, and a pediatric dentist with longitudinal involvement in interdisciplinary CLP teams. Prior to main scoring, raters completed a structured calibration session using five sample responses drawn from a pilot run not included in the final dataset. During calibration, each rater independently assigned GQS scores, after which all three convened to compare ratings and discuss discrepancies. Anchor definitions were clarified iteratively until consensus was reached on each anchor point, with particular attention to distinguishing between scores of 3 and 4 (the most frequent source of initial disagreement) based on the threshold of whether “most important topics” were present with minor inaccuracies versus incomplete coverage of key content. No post-calibration numerical adjustment was applied to main-study scores; raters scored independently throughout.

### 2.4. Statistical Analysis

Analyses were performed in R (version 4.3.2; R Foundation for Statistical Computing, Vienna, Austria) using the irr package (version 0.84.1) for reliability estimation and the tidyverse suite (version 2.0.0) for data management. Inter-rater reliability was assessed for each of the 12 model–domain combinations using Fleiss’ kappa (κ) with associated 95% confidence intervals (CIs) and significance tests against chance agreement [25,26,27]. Kappa values were interpreted as follows: <0.20 = slight, 0.21–0.40 = fair, 0.41–0.60 = moderate, 0.61–0.80 = substantial, and >0.80 = almost perfect agreement [25]. For methodological rigor, domains were included in head-to-head model comparison only if both models achieved substantial agreement (κ ≥ 0.60) [25]. For included domains, a composite score was computed for each response as the mean of the three rater scores (range 1.00–5.00 in 0.33-point increments). Model comparisons used the Wilcoxon signed-rank test for paired ordinal-derived outcomes, with two-sided significance set at *p* < 0.05; the number of positive, negative, and tied pairs is reported alongside each test statistic. Effect sizes were computed as the matched-pairs rank-biserial correlation coefficient (r = 1 − (4V)/(*n*(*n* + 1)), where V is the Wilcoxon statistic and n is the number of non-tied pairs). The Hodges–Lehmann estimator was used to compute 95% CIs for the median paired difference for the overall comparison and for each domain separately. Because domain-specific comparisons were pre-specified as exploratory and the number of testable domains was limited to three by the reliability filter, no multiple-comparison correction was applied; domain-level *p*-values should therefore be interpreted as hypothesis-generating rather than confirmatory.

### 2.5. Qualitative Analysis of Excluded Domains and Error Taxonomy

To address the clinical significance of domains excluded due to low inter-rater reliability, a post hoc qualitative analysis was conducted. Two investigators (A.B. and M.A.) independently reviewed all responses in the During-Treatment, Post-Treatment, and Case Scenario domains and classified disagreements into thematic categories: (a) questions where established clinical guidelines exist but LLM output deviated from them, (b) questions where no consensus guidelines exist, and rater disagreement reflected genuine practice variation, and (c) questions where LLM output contained verifiable factual errors. Discrepancies in classification were resolved by discussion with a third investigator (O.E.).

Additionally, a descriptive taxonomy of errors was developed for the three reliably rated domains. All responses scoring ≤ 3 on the GQS (by ≥2 raters) were reviewed, and specific inaccuracies were categorized into the following types: (i) outdated or incorrect surgical timing, (ii) omission of key multidisciplinary team members, (iii) overgeneralization of outcomes or expectations, (iv) fabricated or unverifiable claims, and (v) incomplete coverage of critical information. The taxonomy was developed using an inductive thematic approach rather than a pre-specified coding framework: two investigators (A.B. and M.A.) independently reviewed the low-scoring responses and generated initial error codes; these were then compared, merged into thematic categories through iterative discussion, and validated against the full response set by a third investigator (O.E.). This process mirrors established qualitative content analysis methodology and is acknowledged to carry the limitations of a small, investigator-generated coding scheme. The GQS itself is a global, holistic measure that does not yield granular, line-by-line factual accuracy scores; the error taxonomy was therefore intended to supplement, not replace, the quantitative GQS analysis.

## 3. Results

### 3.1. Inter-Rater Reliability

**Figure 1:**
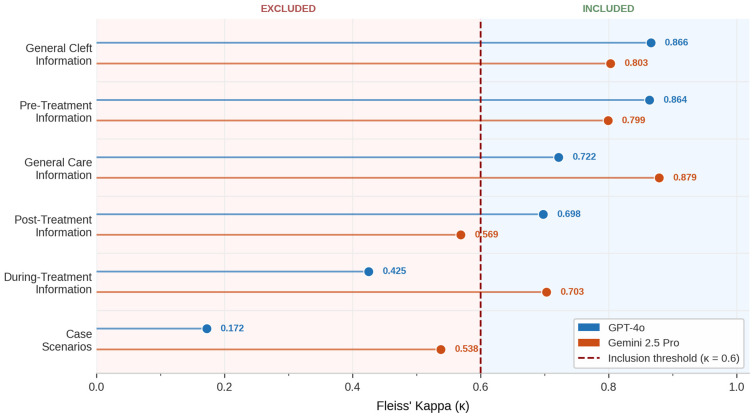
Fleiss’ kappa (κ) inter-rater reliability for GPT-4o and Gemini 2.5 Pro across six CLP information domains. The dashed vertical line marks the κ ≥ 0.60 inclusion threshold. Domains in the blue zone met the threshold for both models and were included in comparative analysis; domains in the red zone were excluded.

Fleiss’ kappa values for all 12 model–domain combinations are presented in Table 1 and Figure 1. Three domains demonstrated substantial to almost perfect inter-rater agreement (κ ≥ 0.60) for both GPT-4o and Gemini 2.5 Pro and were included in the comparative analysis: General Care Information (GPT-4o κ = 0.722; Gemini κ = 0.879), General Cleft Information (GPT-4o κ = 0.866; Gemini κ = 0.803), and Pre-Treatment Information (GPT-4o κ = 0.864; Gemini κ = 0.799). These three domains comprised 30 questions.

### 3.2. Overall Model Comparison

**Figure 2:**
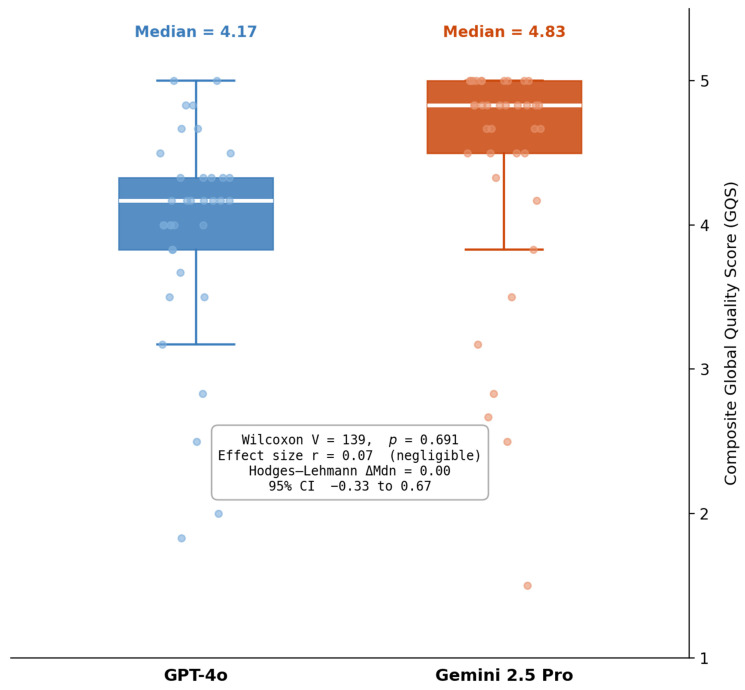
Distribution of composite Global Quality Score (GQS) for GPT-4o and Gemini 2.5 Pro (*n* = 30 paired responses across three reliably rated domains). Boxes represent the IQR; horizontal white line = median; dots = individual responses (jittered for clarity).

The comparative analysis included 30 paired responses from the three reliable domains. Overall, information quality did not differ significantly between models, and the data support practical equivalence rather than merely a failure to detect a difference. GPT-4o achieved a median composite GQS of 4.33 (IQR 4.00–5.00), and Gemini 2.5 Pro achieved a median composite GQS of 5.00 (IQR 4.00–5.00). The Wilcoxon signed-rank test showed no statistically significant difference (V = 139.00, *p* = 0.691; positive pairs = 9, negative pairs = 8, tied pairs = 13; matched-pairs rank-biserial correlation r = 0.07, indicating a negligible effect size). The Hodges–Lehmann estimator for the median paired difference was 0.00 (95% CI: −0.33 to 0.67), confirming that the expected difference between models was zero (Table 2, Figure 2).

### 3.3. Domain-Specific Comparisons

**Figure 3:**
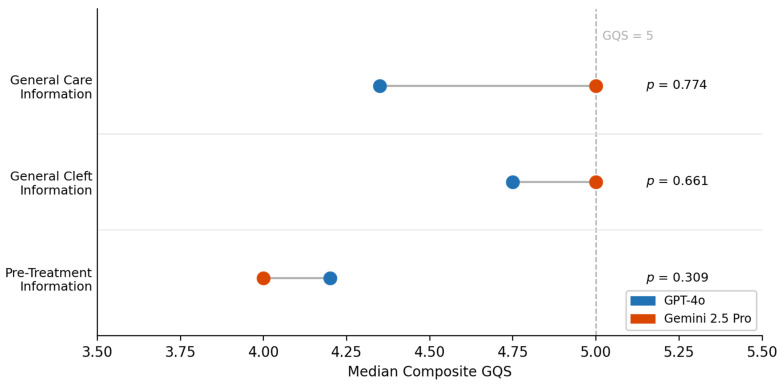
Median composite GQS per model across the three reliably rated domains. Dots represent median scores; connecting lines indicate the paired comparison. *p*-values are from exploratory Wilcoxon signed-rank tests (Bonferroni correction not applied; results are hypothesis-generating).

No significant differences were detected in any reliably evaluated domain; all domain-level analyses should be interpreted as exploratory (Table 3, Figure 3). In General Care Information (10 pairs), Gemini had a higher numerical median score (5.00) than GPT-4o (4.16), but the difference was not significant (V = 22.00, *p* = 0.774; Hodges–Lehmann 95% CI −0.67 to 0.67). In General Cleft Information (10 pairs), Gemini’s median score was 5.00 versus 4.66 for GPT-4o (V = 19.00, *p* = 0.661; 95% CI −0.33 to 0.67). In Pre-Treatment Information (10 pairs), GPT-4o had a slight numerical advantage (median 4.16 vs. 4.00) that was not significant (V = 40.00, *p* = 0.309; 95% CI −0.33 to 1.00). The narrow confidence intervals across all three domains are consistent with practical equivalence of the models in these well-defined, consensus-supported areas.

### 3.4. Qualitative Analysis of Excluded Domains

Qualitative review of the three excluded domains revealed distinct patterns of rater disagreement (Table 4). In the During-Treatment domain, 6 of 10 questions addressed topics where no single consensus guideline exists (e.g., timing of secondary speech surgery, indications for revision rhinoplasty). Rater disagreement predominantly reflected genuine differences in institutional practice rather than factual errors in LLM output. In the post-treatment domain, disagreement centered on questions about long-term prognosis and the definition of treatment completion, topics inherently influenced by individual clinical experience and patient variability.

The Case Scenario domain showed the lowest reliability, particularly for GPT-4o (κ = 0.172). Qualitative review revealed that GPT-4o’s scenario responses tended to be more generic and hedged, providing broadly applicable but non-specific recommendations that raters scored inconsistently. Gemini’s scenario responses were more structured and protocol-oriented but occasionally included recommendations inconsistent with current evidence (e.g., suggesting nasoalveolar molding [NAM] as universally indicated). In both models, the integration of multiple clinical variables required for scenario-based reasoning appeared to exceed the models’ capacity for consistent, clinically appropriate synthesis.

### 3.5. Error Taxonomy in Reliably Rated Domains

**Figure 4:**
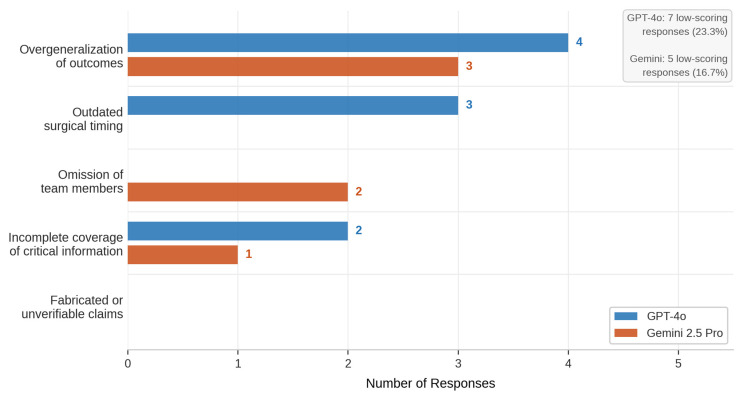
Error taxonomy for responses scoring ≤ 3.0 on the composite GQS (≥2 raters) in the three reliably rated domains. Numbers indicate frequency of each error type per model. Fabricated claims were absent in both models in reliably rated domains.

Among the 60 responses in the three reliable domains (30 per model), 7 GPT-4o responses (23.3%) and 5 Gemini responses (16.7%) received a mean GQS ≤ 3.0. The most common error categories are summarized in Table 5 and Figure 4. Overgeneralization of outcomes (e.g., stating that speech normalizes in all cases after palatoplasty) was the most frequent error type for both models, occurring in 4 GPT-4o responses (57.1% of GPT-4o low-scoring responses) and 3 Gemini responses (60.0% of Gemini low-scoring responses). Outdated surgical timing was observed in 3 GPT-4o responses (42.9%) that referenced older protocols for primary lip repair. Omission of key team members (e.g., failure to mention audiologists or psychologists) occurred in 2 Gemini responses (40.0%). Neither model produced fabricated statistical claims in the reliable domains, although both models occasionally generated hallucinated references when prompted to cite sources during initial pilot testing (these hallucinated citations were corrected during data collection by re-prompting without citation requests). The exclusion of three domains from quantitative comparison should be understood as reflecting limitations in the evaluation framework—particularly the absence of universal clinical consensus in those areas—rather than as evidence of systematic model failure.

## 4. Discussion

This study compared the quality of CLP-related information generated by GPT-4o and Gemini 2.5 Pro using a validated quality instrument and a reliability-first analytic approach. In the three domains where inter-rater agreement was substantial for both models, overall information quality was high (median GQS ≥ 4.0) and did not differ significantly between models. These findings suggest that both LLMs are capable of producing clinically sound general information about CLP that may serve as a useful adjunct for patient and family counseling in consensus-supported domains.

A central methodological contribution of this study is the application of a reliability-first framework, whereby inter-rater agreement was formally assessed as a prerequisite for valid model comparison. This approach departs from much of the existing literature on AI-generated health information, where expert ratings are typically aggregated without verification of rater consistency [6,14,15,16,28]. Our finding that three of six domains failed to achieve adequate reliability for at least one model underscores the importance of this step: comparing models on unreliably rated outcomes would yield uninterpretable results and potentially misleading conclusions.

The pattern of reliability across domains provides clinically meaningful insight. Domains addressing well-established, consensus-supported topics—such as general disease information, multidisciplinary team roles, and pre-treatment counseling—yielded both high-quality LLM outputs and high rater agreement. In contrast, domains involving treatment-specific decisions, postoperative management, and complex clinical scenarios showed lower reliability. These gradients parallel the structure of CLP evidence itself: general principles are well codified in guidelines and textbooks [17,21,22], whereas treatment sequencing, surgical technique selection, and individualized care planning remain variable across institutions and lack universal protocols [22].

The qualitative analysis of excluded domains revealed that low inter-rater reliability was primarily attributable to genuine variation in practice rather than to gross LLM errors. This distinction is important: it suggests that the LLMs did not necessarily perform poorly in these domains but rather that the evaluation framework itself cannot reliably discriminate quality when expert opinion is divided. Future studies may benefit from evaluating treatment-specific LLM outputs against institution-specific protocols rather than relying on inter-rater agreement among clinicians from potentially different practice traditions.

The descriptive error taxonomy identified overgeneralization as the predominant error type in both models. LLMs tended to present treatment outcomes in overly optimistic terms without acknowledging variability, which could create unrealistic expectations for families. Outdated surgical timing recommendations were also observed, consistent with the known training-data lag inherent in LLMs. These findings align with prior observations in oral surgery [5,14] and otolaryngology [6], where LLMs have been noted to provide plausible but imprecise clinical information. Importantly, neither model fabricated clinical statistics in the reliably rated domains, although hallucinated references were encountered during pilot testing—a well-documented limitation of current LLMs [8,9].

Our finding that both models achieved high median GQS scores in reliable domains is consistent with recent studies evaluating LLM performance in adjacent fields. Jacobs et al. [14] reported that ChatGPT provided accurate and readable information for patients undergoing third molar extraction, and Cai et al. [16] found GPT-4 to be largely appropriate for post-surgical follow-up guidance. A recent study by Acar et al. [28] similarly demonstrated acceptable LLM performance on oral surgery consultations in consensus-supported clinical domains. Recent implant-dentistry literature further supports this domain-dependent interpretation: Neji et al. described AI-powered predictive models as increasingly relevant to implant planning, risk assessment, surgical navigation, peri-implant disease monitoring, and outcome prediction, while emphasizing that clinical integration still requires validation, clinician oversight, and careful translation into decision-making workflows [29]. However, direct comparisons across studies are limited by differences in question design, rating instruments, model versions, and evaluation methodologies. The present study’s use of a split-mouth-style paired design (same questions to both models) and formal reliability gating strengthens internal validity relative to single-model or unpaired evaluations. The broader implications of these findings extend beyond CLP care. The domain-reliability gradient observed in this study—whereby consensus-codified topics yielded both high-quality LLM outputs and high inter-rater agreement, whereas practice-variable and scenario-based domains did not—likely applies across any complex, multidisciplinary clinical pathway. Conditions such as craniofacial syndromes, pediatric oncology, and congenital cardiac disease share the same structural feature: a well-defined body of general knowledge alongside highly individualized, center-specific treatment protocols. Clinicians and health information designers in these fields may find the reliability-first analytic framework transferable and are encouraged to adopt it before reporting aggregate quality scores for AI-generated health content. At a system level, the finding that LLM quality is selectively high underscores that wholesale acceptance or wholesale rejection of AI-generated health information is equally inappropriate; what is needed are domain-specific validation standards and transparent communication tools that help clinicians and patients navigate the boundary between reliable and unreliable AI output.

From a clinical perspective, these data support a tiered approach to LLM use in CLP care. This can be operationalized through a straightforward framework distinguishing appropriate from inappropriate clinical use. Appropriate uses include: directing families to use LLMs as a supplement to specialist counseling for understanding general CLP etiology, prevalence, and inheritance patterns; explaining the composition of the multidisciplinary team and the rationale for each specialty’s involvement; and answering broad pre-treatment questions such as general feeding strategies, the expected sequence of interventions, or the availability of support groups. To illustrate, a parent who asks an LLM “What specialists will be involved in my child’s cleft care?” is likely to receive an accurate, high-quality response consistent with what a CLP nurse coordinator might provide. Inappropriate uses include relying on LLM-generated guidance for decisions about surgical timing (e.g., optimal age for primary palatoplasty or secondary alveolar bone grafting), interpreting specific postoperative complication signs without clinical context, or using LLM scenario-based reasoning to guide individualized treatment planning. In these domains, LLM outputs in the present study showed either insufficient inter-rater reliability for objective scoring or errors such as referencing outdated surgical protocols. For example, an LLM response stating that speech outcomes are routinely normalized after palatoplasty may provide false reassurance and should be corrected proactively by the clinician. A practical recommendation is that CLP team coordinators or speech-language pathologists develop a short written “LLM caveat sheet”—a one-page summary of topics for which AI information is generally trustworthy versus topics that require direct team input—which can be provided to newly diagnosed families alongside standard clinic materials. This tiered framework is consistent with emerging recommendations for responsible AI use in healthcare [23,24,29] and extends them with domain-specific evidence from a complex surgical subspecialty.

This study has several limitations. First, LLM outputs represent a snapshot of model performance on 25 September 2024; subsequent model updates may alter output quality in either direction. This reinforces the need for longitudinal re-evaluation studies that periodically re-administer the same question sets to updated model versions, allowing temporal tracking of accuracy trends as LLMs are retrained or fine-tuned. Second, each question was submitted only once per model; repeated submissions could yield different responses due to the stochastic nature of language model generation, and test–retest variability was not characterized. Third, the GQS, while validated and widely used, assesses overall quality holistically rather than providing granular, line-by-line factual accuracy scoring; the error taxonomy was developed to partially compensate for this limitation but remains investigator-generated and should not be treated as a validated instrument. Fourth, the question set, although developed by experienced clinicians and piloted for lay comprehensibility, may not fully capture the diversity of patient phrasing, languages, cultural contexts, and health literacy levels encountered in real-world information-seeking. No patient or lay public input was incorporated into question development, which is a meaningful gap, given that the primary intended end users of AI-generated CLP information are families rather than clinicians. Fifth, the study did not differentiate between healthcare-professional and lay-user contexts: a clinician querying the same question as a family caregiver may receive an identical response, but the relevance, comprehensibility, and potential for harm of that response differ substantially across user backgrounds. Future studies should explicitly stratify analyses by intended user type. Sixth, reliability was evaluated within a single institutional rater panel; raters from different geographic or institutional contexts may apply GQS anchors differently, particularly in domains where practice variation is high. Seventh, the sample of 54 questions, while broad in thematic scope, limits the statistical power to detect small between-model differences. Eighth, the study did not evaluate readability (e.g., Flesch–Kincaid grade level), health literacy alignment, cultural sensitivity, or patient comprehension outcomes—all of which are central to the effectiveness of patient-facing health information and represent important directions for future research. Ninth, ethical dimensions of LLM use in clinical contexts—including data privacy, the absence of informed consent for AI-generated health consultations, the allocation of clinical responsibility when patients act on AI advice, and the risk of exacerbating health disparities if AI tools are disproportionately accessed by more health-literate populations—were beyond the scope of this study but warrant explicit consideration in guidelines for AI integration in CLP care. Finally, the external generalizability of the findings to other complex chronic conditions managed by multidisciplinary teams (e.g., craniofacial syndromes, oncologic conditions, congenital cardiac disease) is plausible based on the observed domain-reliability gradient, but requires empirical confirmation in those fields.

## 5. Conclusions

Both GPT-4o and Gemini 2.5 Pro generated high-quality information on cleft lip and palate in domains supported by clinical consensus, with no statistically significant or practically meaningful differences between models. Treatment-specific and scenario-based domains showed insufficient inter-rater reliability for valid comparison, reflecting both the inherent practice variability in CLP care and the limitations of current LLMs in synthesizing complex, individualized clinical reasoning. A descriptive error taxonomy identified overgeneralization of outcomes, outdated surgical timing, and incomplete multidisciplinary team descriptions as the most common inaccuracy types. For clinical practice, these findings translate into a directly actionable recommendation: LLMs may be safely directed toward general CLP education tasks—explaining disease etiology, the structure of the multidisciplinary team, the broad sequence of interventions, and pre-treatment preparation—while clinicians should proactively advise families that AI-generated content on surgical timing decisions, postoperative complication management, and individualized treatment planning requires verification against institutional protocols. CLP teams are encouraged to develop brief, family-facing guidance documents that delineate which AI-generated information is generally trustworthy and which should prompt direct contact with a clinician. Platforms delivering AI-generated health information in CLP contexts should implement domain-specific quality warnings to alert end users when entering practice-variable territory. Future research priorities should include: (i) periodic re-evaluation of LLM outputs as models are updated, to track accuracy improvements over time; (ii) benchmarking of AI-generated content against established clinical practice guidelines on a line-by-line basis; (iii) assessment of patient and caregiver comprehension of AI-generated CLP information across diverse health literacy levels; and (iv) prospective investigation of whether clinician-mediated use of LLM-generated materials improves family preparedness and satisfaction compared with standard written information alone.

## Figures and Tables

**Table 1:** Inter-rater reliability (Fleiss’ kappa) by domain and model.

Domain	GPT-4o κ	Gemini 2.5 Pro κ	Included (κ ≥ 0.60 Both)
General Care Information	0.722	0.879	Yes
General Cleft Information	0.866	0.803	Yes
Pre-Treatment Information	0.864	0.799	Yes
During-Treatment Information	0.425	0.703	No
Post-Treatment Information	0.698	0.569	No
Case Scenarios	0.172 (*p* = 0.45)	0.538	No

**Table 2:** Overall quality comparison across reliably evaluated domains (*n* = 30 paired questions).

Model	Median GQS	IQR	Wilcoxon V	*p* Value
GPT-4o	4.33	4.00–5.00	139.00	0.691
Gemini 2.5 Pro	5.00	4.00–5.00	—	—

95% CI for median paired difference: −0.33 to 0.67.

**Table 3:** Domain-specific comparisons for reliably evaluated domains.

Domain	GPT-4o Median GQS	Gemini 2.5 Pro Median GQS	*p* Value
General Care Information	4.16	5.00	0.774
General Cleft Information	4.66	5.00	0.661
Pre-Treatment Information	4.16	4.00	0.309

**Table 4:** Qualitative analysis of domains excluded due to low inter-rater reliability.

Domain	Primary Reason for Low κ	Representative Finding
During-Treatment	Practice variation (6/10 questions lacked consensus guidelines)	Raters disagreed on appropriateness of speech surgery timing recommendations
Post-Treatment	Subjective long-term prognosis assessment	No consensus on when treatment is “definitively complete”
Case Scenarios	Generic/hedged outputs (GPT-4o); protocol inconsistencies (Gemini)	GPT-4o: non-specific recommendations; Gemini: NAM suggested as universal

**Table 5:** Error taxonomy in reliably rated domains.

Error Category	GPT-4o (n)	Gemini 2.5 Pro (n)
Overgeneralization of outcomes	3	3
Outdated surgical timing	3	1
Omission of key team members	1	2
Fabricated/unverifiable claims	0	0
Incomplete coverage of critical information	2	1

Note: Counts refer to responses with mean GQS ≤ 3.0 by ≥2 raters. Some responses contained multiple error types.

## Data Availability

The complete list of 54 questions is provided in Appendix A. Representative questions with corresponding AI-generated responses from both models are included in Supplementary Material S1. Individual rater scores and composite GQS values for all 108 responses are provided as Supplementary Materials S2. The full AI-generated response dataset, raw rater scores, and exclusion decisions are available from the corresponding author upon reasonable request.

## Supplementary Materials

The following supporting information can be downloaded at: https://www.mdpi.com/article/10.3390/healthcare14111535/s1, Supplementary Materials S1: Representative sample of questions with corresponding AI-generated responses from GPT-4o and Gemini 2.5 Pro, including instances where responses were incomplete on first submission. Supplementary Materials S2: Individual rater scores, composite GQS values, and domain-level exclusion decisions for all 108 responses.

## Appendix A. Complete Question Set

Group 1: General Cleft Information (10 questions)

1. What is cleft lip and cleft palate?
2. What causes cleft lip and cleft palate?
3. Are cleft lip and cleft palate hereditary?
4. How common are cleft lip and cleft palate?
5. Can cleft lip and cleft palate be diagnosed before birth?
6. What are the possible effects of cleft lip and cleft palate on my child?
7. Are there different types of cleft lip and cleft palate? If so, what are they?
8. Will this condition affect my child’s ability to eat or speak?
9. Does this condition affect hearing?
10. Which medical professionals will be involved in my child’s care?

Group 2: Pre-Treatment Information (10 questions)

1. When is the first surgery to repair the lip usually recommended?
2. When is surgery to repair the palate usually recommended?
3. How should I feed my baby before surgery?
4. What are the possible risks and complications of the initial surgeries?
5. What will recovery after surgery look like?
6. How long will my child stay in the hospital after surgery?
7. Will there be scars after surgery?
8. Will my child need additional treatment after the initial surgery?
9. What kind of preparation is needed before surgery?
10. Are there support groups for parents in a similar situation?

Group 3: During-Treatment Information (10 questions)

1. My child has had lip surgery and still has feeding difficulties. Is this normal?
2. When should I expect improvement in my child’s speech after palate surgery?
3. My child is 3 years old and has already had two surgeries. Will more be needed?
4. Is speech therapy necessary in all cases?
5. At what age should orthodontic treatment begin?
6. What signs should concern me after surgery?
7. How should I clean the surgical area?
8. Are there any restrictions on physical activity during the recovery period?
9. Is there a possibility of additional surgeries for aesthetic or functional correction?
10. What should I do if there is an infection in the surgical area?

Group 4: Post-Treatment Information (10 questions)

1. My child has completed all surgeries. Does the risk of complications decrease?
2. Is there still a risk of speech problems after treatment is completed?
3. Will my child still need regular follow-up examinations in adulthood?
4. Is psychological support available for older children and adolescents with cleft lip and palate?
5. Can the scars left after surgery be removed?
6. Will my child be able to live a completely normal life?
7. What should I know about future pregnancy in relation to cleft lip and palate?
8. Are there late complications that may develop?
9. Will my child need special dental treatment in the future?
10. When can it be said that treatment is definitively complete?

Group 5: General Care Information (10 questions)

1. What is the role of a multidisciplinary team in treatment?
2. Which medical specialties are involved in treatment?
3. What is the importance of orthodontic treatment in cleft lip and palate care?
4. What is the role of speech-language pathologists in the process?
5. Are there any complementary or alternative treatments?
6. What are the costs involved in treatment, and are they covered by insurance?
7. How do you manage feeding challenges in infants with cleft lip and palate?
8. What is the importance of genetic counseling in these cases?
9. Are there any new studies or breakthroughs in treatment?
10. How is the patient’s medical record maintained over the many years of treatment?

Group 6: Case Scenarios (4 scenarios)

1. Case Scenario 1: Isolated cleft palate, before surgery
2. Case Scenario 2: Unilateral cleft lip, after surgery
3. Case Scenario 3: Complete cleft lip and palate, speech difficulties at a young age
4. Case Scenario 4: Long-term follow-up, need for additional treatments
