# Supplementary material for: Evaluating the Quality of Artificial Intelligence-Generated Information on Cleft Lip and Palate: A Comparative Cross-Sectional Study
Source: Healthcare (Basel). 2026 Jun 1;14(11):1535. doi: 10.3390/healthcare14111535 (PMC13257001; doi:10.3390/healthcare14111535)
Supplement: Supplementary file 1 [file healthcare-14-01535-s001.zip › Supplementary_S1_QA_Pairs.pdf]

SUPPLEMENTARY MATERIALS S1

Representative AI-Generated Responses to CLP Questions

GPT-4o (OpenAI) vs. Gemini 2.5 Pro (Google DeepMind)

Data Collection Date: 25 September 2024

Notes to readers: All 54 questions are presented below, organized by domain. Responses are verbatim outputs from each model. Domains marked [INCLUDED] met the κ ≥ 0.60 inter-rater reliability threshold for both models and were included in comparative statistical analysis. Domains marked [EXCLUDED] were retained for qualitative analysis only. Responses flagged with ⚑ resubmitted were incomplete or declined on first submission and were re-submitted once in a new session (n = 3 total across both models: Q51-GPT, Q53-GPT, Q54-Gemini). The flag symbol and pink shading identify these cells.

General Cleft Information **[INCLUDED** — κ(GPT-4o) = 0.866, κ(Gemini) = 0.803]

Q1. What are cleft lip and cleft palate?

GPT-4o	Gemini 2.5 Pro
Cleft lip and cleft palate are congenital craniofacial malformations caused by incomplete fusion of the lip and/or the roof of the mouth during early fetal development. A cleft lip appears as a split in the upper lip, while a cleft palate is an opening in the hard or soft palate. They may occur separately or together, on one or both sides.	Cleft lip and cleft palate are birth defects in which the upper lip and/or the palate do not fuse completely during pregnancy. The result is a visible gap in the lip, the palate, or both. They are among the most common congenital anomalies of the face and mouth.

Q2. What causes cleft lip and cleft palate?

GPT-4o	Gemini 2.5 Pro
The etiology is multifactorial, involving both genetic and environmental factors. Contributing factors include family history, maternal smoking, alcohol use, certain medications (e.g., anticonvulsants), folic	The exact cause is often unknown. It is believed to result from a combination of genetic predisposition and environmental influences such as smoking, alcohol consumption, poor nutrition, and certain medications taken during early pregnancy.

acid deficiency, and uncontrolled diabetes during pregnancy.	

**Q3.** Are cleft lip and cleft palate hereditary?	
**GPT-4o**	**Gemini 2.5 Pro**
There is a clear genetic component. If a parent or sibling has a cleft, the risk in subsequent children is increased. Some clefts are part of recognized genetic syndromes (e.g., Van der Woude, 22q11.2 deletion), while most are nonsyndromic with complex inheritance.	Yes, heredity can play a role. The risk is higher when there is a family history of cleft. However, many cases occur in families with no previous history, indicating that environmental factors also contribute.

**Q4.** How common is the occurrence of cleft lip and cleft palate?	
**GPT-4o**	**Gemini 2.5 Pro**
Globally, cleft lip with or without cleft palate occurs in approximately 1 in 700 live births. Incidence varies by ethnicity: highest among Asian and Native American populations, lower among individuals of African descent.	Clefts are among the most common congenital anomalies, affecting roughly 1 in every 700 newborns worldwide. The rate differs by region and ethnic background.

**Q5.** Can cleft lip and cleft palate be diagnosed before birth?	
**GPT-4o**	**Gemini 2.5 Pro**
Yes. Cleft lip can typically be identified by routine prenatal ultrasound from around 18–22 weeks of gestation. Isolated cleft palate is harder to detect prenatally and is often diagnosed at birth.	Cleft lip is frequently visible on prenatal ultrasound, especially in the second trimester. Cleft palate alone is more difficult to see before birth and is usually confirmed during the newborn examination.

**Q6.** What are the potential implications of cleft lip and cleft palate for my child?	
**GPT-4o**	**Gemini 2.5 Pro**

Possible implications include feeding difficulties, speech and language delays, recurrent ear infections and hearing loss, dental and orthodontic problems, and psychosocial challenges. With coordinated multidisciplinary care, most children achieve excellent outcomes.	Children with cleft may face challenges with feeding, speech, hearing, and dental development. Some may also experience emotional or social difficulties. Comprehensive treatment usually minimizes these effects.

**Q7.** Are there different types of cleft lip and cleft palate? If so, what are they?	
**GPT-4o**	**Gemini 2.5 Pro**
Yes. Clefts are classified by location and extent: cleft lip only, cleft palate only, or combined cleft lip and palate. They can be unilateral or bilateral, and complete or incomplete. A submucous cleft palate is a hidden form where the muscle is split beneath intact mucosa.	There are several types: cleft lip alone, cleft palate alone, and combined cleft lip and palate. Each can be unilateral (one side) or bilateral (both sides), and complete or incomplete in severity.

**Q8.** Will the condition affect my child's ability to eat or speak?	
**GPT-4o**	**Gemini 2.5 Pro**
Initially, feeding can be challenging due to difficulty creating suction, and specialized bottles are often needed. Speech may be affected, particularly with cleft palate, but most children develop normal or near-normal speech following surgical repair and speech therapy.	Yes, eating and speaking may be affected before treatment. With specialized feeding techniques, surgery, and speech therapy, most children eat normally and develop clear speech.

**Q9.** Does this condition affect hearing?	
**GPT-4o**	**Gemini 2.5 Pro**
Yes. Children with cleft palate are prone to Eustachian tube dysfunction and recurrent middle ear effusions, which can cause conductive hearing loss. Regular ENT and audiology follow-up, and often ventilation tubes, are recommended.	Hearing can be affected, especially in children with cleft palate, because of recurrent middle ear infections. Routine hearing tests and ENT monitoring are an important part of care.

**Q10.** Which medical professionals will be involved in my child's care?	
GPT-4o	Gemini 2.5 Pro
A multidisciplinary cleft team typically includes a plastic/craniofacial surgeon, oral and maxillofacial surgeon, ENT, pediatrician, orthodontist, pediatric dentist, speech-language pathologist, audiologist, geneticist, psychologist, social worker, and nursing/feeding specialists.	Care is delivered by a multidisciplinary team that usually includes surgeons, ENT specialists, dentists, orthodontists, speech therapists, audiologists, geneticists, psychologists, and nurses who coordinate treatment from infancy to adulthood.

Pre-Treatment Information [INCLUDED — κ(GPT-4o) = 0.864, κ(Gemini) = 0.799]

**Q11.** When is it recommended to perform the first surgery to repair the lip?	
GPT-4o	Gemini 2.5 Pro
Primary cleft lip repair is typically performed between 3 and 6 months of age, often following the 'rule of 10s' (10 weeks old, 10 pounds, hemoglobin >10 g/dL).	Cleft lip repair is usually done between 3 and 6 months of age, once the baby is healthy and gaining weight appropriately.

**Q12.** When is it recommended to perform the surgery to repair the palate?	
GPT-4o	Gemini 2.5 Pro
Cleft palate repair (palatoplasty) is generally performed between 9 and 18 months of age to optimize speech development while minimizing impact on midface growth.	Palate repair is typically performed between 9 and 18 months, prior to the development of speech, to support normal speech outcomes.

**Q13.** How should I feed my baby before the surgery?	
GPT-4o	Gemini 2.5 Pro
Specialized bottles and nipples (e.g., Haberman, Pigeon, Dr. Brown's Specialty Feeder) are used to	Babies with cleft usually need special bottles designed for cleft feeding. Hold the baby upright,

compensate for the inability to generate suction. Upright positioning, frequent burping, and paced feeding help reduce air swallowing and aspiration.	feed slowly, and burp frequently. A feeding specialist or nurse can guide you on the best technique.

Q14. What are the potential risks and complications of the initial surgeries?

GPT-4o	Gemini 2.5 Pro
Possible risks include bleeding, infection, anesthesia-related complications, wound dehiscence, fistula formation (especially after palatoplasty), scarring, and the potential need for revision surgery. Overall, these procedures are considered safe in experienced hands.	As with any operation, risks include bleeding, infection, reaction to anesthesia, scarring, and occasionally the need for additional surgery. Serious complications are uncommon when performed by experienced cleft teams.

Q15. What will the recovery from surgery look like?

GPT-4o	Gemini 2.5 Pro
Recovery typically involves a short hospital stay, pain management, soft-diet or syringe/spoon feeding for a few weeks, arm restraints to prevent wound disturbance, and careful wound care. Most children resume normal activity within 2–4 weeks.	After surgery, expect some swelling and discomfort, modified feeding for a couple of weeks, and careful wound care. Most babies recover quickly and return to normal routines within a few weeks.

Q16. How long will my child stay in the hospital after the surgery?

GPT-4o	Gemini 2.5 Pro
Most children remain hospitalized for 1–3 days after lip or palate repair, depending on feeding, pain control, and any complications.	The hospital stay is generally 1–3 days, depending on how well the child eats and recovers after the procedure.

Q17. Will there be scars after the surgery?

GPT-4o	Gemini 2.5 Pro

Yes, a small scar along the lip is expected, but modern surgical techniques (e.g., Millard or Fisher repair) and scar management usually result in a subtle, well-positioned scar that fades significantly over time.	There will be a scar on the lip, but it typically becomes less noticeable with time. Massage, sun protection, and occasionally revision surgery can further improve appearance.

Q18. Will my child need additional treatment after the initial surgery?

GPT-4o	Gemini 2.5 Pro
Yes. Many children require additional procedures over the years, including alveolar bone grafting (around 7–11 years), orthodontics, speech therapy, ENT procedures (e.g., ear tubes), and sometimes lip/nose revision or orthognathic surgery in adolescence.	Yes, treatment is usually long-term. It may include orthodontics, speech therapy, ear tube placement, dental care, bone grafting, and occasionally revision surgeries during childhood and adolescence.

Q19. What kind of preparation is required before the surgery?

GPT-4o	Gemini 2.5 Pro
Preparation includes preoperative pediatric and anesthesia evaluation, blood work, optimization of weight and hemoglobin, ensuring the child is free of acute illness, fasting per anesthesia guidelines, and reviewing post-op feeding and care plans with the family.	Before surgery, the baby is examined to confirm good general health, weight gain, and the absence of infection. Parents receive instructions about fasting, medications, and what to expect on the day of surgery.

Q20. Are there support groups for parents in a similar situation?

GPT-4o	Gemini 2.5 Pro
Yes. Many cleft centers offer parent support groups, and several national and international organizations (e.g., Cleft Lip and Palate Association, AmeriFace, Smile Train, Operation Smile) provide peer support, information, and online communities.	Yes. Local hospitals and national organizations often run support groups and online communities for families. Connecting with other parents can be very helpful emotionally and practically.

During-Treatment Information [EXCLUDED — κ(GPT-4o) = 0.425, κ(Gemini) = 0.703]

Q21. My child had lip surgery and still has eating difficulties; is this normal?

GPT-4o	Gemini 2.5 Pro
Mild feeding difficulties for a few weeks after lip repair are common due to swelling, tenderness, and changes in suckling mechanics. Persistent or worsening problems should be evaluated by the cleft team or a feeding specialist.	Some feeding difficulties after lip surgery are normal during the healing period. If problems continue beyond a few weeks or your baby is not gaining weight, contact the cleft team for evaluation.

Q22. When should I expect an improvement in my child's speech after palate surgery?

GPT-4o	Gemini 2.5 Pro
Speech typically begins to develop normally after palate repair, but noticeable improvement may take months to years. Speech therapy is often required, and progress is monitored regularly. Some children may need additional procedures for velopharyngeal insufficiency.	Improvement in speech is gradual and may take months or even a few years. Many children benefit from speech therapy, and progress is reviewed regularly by the speech-language pathologist.

Q23. My child is 3 years old and has already undergone two surgeries; will they need more?

GPT-4o	Gemini 2.5 Pro
Possibly. Common future procedures include alveolar bone grafting around ages 7–11, lip/nose revision, ear tube placement, speech-related surgery if needed, and orthognathic surgery in adolescence. The exact plan depends on the type and severity of the cleft.	Children with cleft often need additional procedures as they grow, such as bone grafting, dental and orthodontic treatment, and sometimes revision surgery. The cleft team will plan each step based on your child's development.

Q24. Are speech therapy treatments necessary in all cases?

GPT-4o	Gemini 2.5 Pro

Not all children require speech therapy, but it is frequently recommended, especially after cleft palate repair. Early evaluation by a speech-language pathologist allows therapy to start as soon as it is indicated.	Not every child needs speech therapy, but many do, particularly those with cleft palate. Early evaluation helps determine if therapy is needed and ensures timely intervention.

Q25. At what age is it advisable to start orthodontic treatment?

GPT-4o	Gemini 2.5 Pro
Initial orthodontic evaluation may begin around age 6–7, with early-phase treatment to prepare for alveolar bone grafting (typically 7–11 years). Comprehensive orthodontics generally starts in adolescence after permanent teeth have erupted.	Orthodontic monitoring often starts around age 6–7. Active treatment usually begins before bone grafting and continues in stages through the teenage years.

Q26. What are the signs that should concern me after the surgery?

GPT-4o	Gemini 2.5 Pro
Warning signs include high fever, increasing pain, swelling, redness, foul-smelling discharge, persistent bleeding, wound separation, breathing difficulties, or poor feeding. Any of these warrant prompt contact with the surgical team.	Contact your doctor if you notice fever, heavy bleeding, increasing pain, foul discharge, opening of the wound, breathing problems, or refusal to eat. These may indicate infection or another complication.

Q27. How should I clean the surgical area?

GPT-4o	Gemini 2.5 Pro
Follow the surgeon's specific instructions. Generally, gentle cleansing with saline or sterile water after feedings, avoiding rubbing, and keeping the area dry is recommended. Avoid hard or sharp foods and pacifiers as advised.	Usually the area is cleaned gently with saline or sterile water after feeds, avoiding pressure or rubbing. Follow the specific written instructions provided by your surgical team.

Q28. Are there restrictions on physical activity for my child during the recovery period?

GPT-4o	Gemini 2.5 Pro
Yes. For 2–4 weeks post-op, children should avoid putting objects in the mouth, rough play, climbing, and contact sports. Arm restraints are often used in infants to prevent disturbing the wound.	Strenuous activity, contact sports, and putting objects in the mouth should be avoided for several weeks after surgery. Your team will advise when normal play can resume.

Q29. Is there a possibility of repeat surgeries for aesthetic or functional correction?

GPT-4o	Gemini 2.5 Pro
Yes. Revision surgeries are common and may address lip and nose appearance, palate function (e.g., velopharyngeal insufficiency), fistula closure, scar improvement, or jaw alignment, depending on the child's needs.	Yes. Many patients have additional surgeries over time to improve appearance, breathing, speech, or bite. These are planned according to the child's growth and individual needs.

Q30. What should I do if there is an infection in the surgical area?

GPT-4o	Gemini 2.5 Pro
Contact the surgical team promptly. Signs include fever, increasing redness, swelling, pain, or pus. Treatment usually involves antibiotics, sometimes wound care or drainage, and close follow-up.	If you suspect infection (redness, swelling, fever, discharge), contact the cleft team or surgeon without delay. Early treatment with antibiotics and proper wound care usually resolves the problem.

Post-Treatment Information [EXCLUDED — κ(GPT-4o) = 0.698, κ(Gemini) = 0.569]

Q31. My child has finished all surgeries; do the risks of complications decrease?

GPT-4o	Gemini 2.5 Pro
Yes, the risk of surgical complications decreases substantially once the planned procedures are completed. However, long-term follow-up remains important for speech, dental, ENT, and psychosocial issues.	Yes. After completing the main surgeries, surgical risks are minimal. Ongoing follow-up is still recommended to monitor speech, hearing, dental, and overall development.

**Q32.** Is there still a risk of speech problems after completing the treatment?	
**GPT-4o**	**Gemini 2.5 Pro**
Some children may continue to have residual speech issues, such as hypernasality or articulation errors, even after all surgeries. Continued speech therapy and, in some cases, additional procedures can address these problems.	Speech problems are possible even after treatment is finished. Continued speech therapy and, if needed, further evaluation can help correct any remaining issues.

**Q33.** Will my child need regular follow-up checks even in adulthood?	
**GPT-4o**	**Gemini 2.5 Pro**
Yes. Periodic follow-up is recommended into adulthood, focusing on dental and orthodontic stability, speech, ENT health, psychosocial wellbeing, and any late functional or aesthetic concerns.	Yes. Long-term follow-up is recommended into adulthood to monitor dental, speech, hearing, and aesthetic outcomes and to address any new issues that may arise.

**Q34.** Is there psychological support available for children and adolescents with cleft lip and palate as they grow up?	
**GPT-4o**	**Gemini 2.5 Pro**
Yes. Most cleft teams include psychologists or social workers who provide support around self-image, peer relationships, school, and any anxiety or low self-esteem related to the condition.	Yes. Psychological support is an important part of cleft care. Counseling and peer support help children and teens cope with appearance, social situations, and self-confidence.

**Q35.** Can the scars remaining after the surgeries be removed?	
**GPT-4o**	**Gemini 2.5 Pro**
Scars cannot be completely removed, but their appearance can often be improved with scar revision surgery, dermabrasion, laser therapy, silicone treatments, and good sun protection during healing.	Scars usually fade over time and can be improved further with massage, silicone products, laser treatments, or revision surgery, although they cannot be fully erased.

**Q36.** Will my child be able to lead a completely normal life?	
**GPT-4o**	**Gemini 2.5 Pro**
Yes. With modern multidisciplinary treatment, the vast majority of children with cleft lip and/or palate lead full, normal lives in terms of education, social relationships, career, and family.	Absolutely. Most children with cleft grow up to lead healthy, normal lives, with no significant limitations in education, work, relationships, or activities.

**Q37.** What should I know regarding future pregnancies in the context of cleft lip and palate?	
**GPT-4o**	**Gemini 2.5 Pro**
The recurrence risk is slightly increased, especially if one parent or sibling is affected. Genetic counseling is recommended, along with optimization of maternal health (folic acid supplementation, avoidance of smoking and alcohol) before and during pregnancy.	The risk of having another child with a cleft is slightly higher than in the general population. Genetic counseling and good prenatal care (folic acid, avoiding smoking and alcohol) are recommended.

**Q38.** Are there late complications that can develop?	
**GPT-4o**	**Gemini 2.5 Pro**
Possible late issues include velopharyngeal insufficiency, oronasal fistulas, midface hypoplasia, malocclusion, dental anomalies, hearing problems, and persistent psychosocial concerns. These are managed by the cleft team as they arise.	Yes. Late issues can include speech changes, dental and bite problems, hearing changes, and aesthetic concerns. Ongoing follow-up allows early identification and treatment.

**Q39.** Will my child need special dental treatments in the future?	
**GPT-4o**	**Gemini 2.5 Pro**
Likely yes. Children with cleft commonly require orthodontics, restorative dentistry for missing or malformed teeth (often the lateral incisor), and sometimes dental implants or prosthetics in adulthood.	Yes. Dental care often includes orthodontics, fillings or crowns for affected teeth, and sometimes implants or bridges later in life. Regular dental check-ups are essential.

**Q40.** When can it be said that the treatment has officially ended?	
**GPT-4o**	**Gemini 2.5 Pro**
'Completion' usually refers to the end of active surgical and orthodontic treatment, typically in late adolescence or early adulthood after final orthognathic, dental, and any aesthetic procedures. Periodic follow-up may continue beyond that point.	Treatment is generally considered complete in late adolescence or early adulthood, once facial growth is done and all planned surgical, orthodontic, and dental procedures are finished.

General Care Information [INCLUDED — κ(GPT-4o) = 0.722, κ(Gemini) = 0.879]

**Q41.** What is the role of a multidisciplinary team in the treatment?	
**GPT-4o**	**Gemini 2.5 Pro**
A multidisciplinary team coordinates all aspects of care from infancy to adulthood, ensuring that surgery, speech, hearing, dental, psychosocial, and developmental needs are addressed in a timely and integrated way, improving overall outcomes.	The team ensures that all medical, surgical, dental, speech, and psychological aspects of cleft care are coordinated. This integrated approach leads to better long-term outcomes.

**Q42.** Which medical specialties are involved in the care?	
**GPT-4o**	**Gemini 2.5 Pro**
Typical specialties include plastic and craniofacial surgery, oral and maxillofacial surgery, ENT, pediatrics, orthodontics, pediatric dentistry, speech-language pathology, audiology, genetics, psychology, and social work.	Care usually involves plastic surgeons, ENT doctors, dentists, orthodontists, speech therapists, audiologists, geneticists, pediatricians, psychologists, and nurses.

**Q43.** What is the importance of orthodontic treatment in cleft lip and palate?	
**GPT-4o**	**Gemini 2.5 Pro**

Orthodontics is essential for aligning teeth, preparing the maxilla for bone grafting, correcting crossbites and arch deficiencies, and optimizing function and aesthetics. It is delivered in stages from childhood through adolescence.	Orthodontic treatment helps correct bite and tooth alignment that are often affected by clefts. It is typically performed in phases, in coordination with surgery, to achieve good function and appearance.

Q44. What is the role of speech-language pathologists in the process?

GPT-4o	Gemini 2.5 Pro
Speech-language pathologists evaluate and treat articulation, resonance, language, and feeding difficulties. They monitor speech development, provide therapy, and help determine whether additional surgical interventions for velopharyngeal function are needed.	Speech-language pathologists assess and treat speech, language, and feeding problems. They help children develop clear speech and identify when additional interventions are needed.

Q45. Are there complementary or alternative treatments?

GPT-4o	Gemini 2.5 Pro
Complementary approaches (e.g., feeding support, psychological counseling, peer support groups) can be beneficial alongside standard care. There are no proven alternative treatments that replace surgical and team-based management of clefts.	Standard cleft treatment is medical and surgical, but supportive approaches such as counseling, nutritional support, and parent groups can complement care. Alternative therapies should always be discussed with the medical team.

Q46. What are the costs involved in the treatment, and are they covered by insurance?

GPT-4o	Gemini 2.5 Pro
Costs are significant due to multiple surgeries, orthodontics, and long-term follow-up. In many countries, cleft care is covered by national health systems or insurance. Coverage varies; social workers or case managers can help families navigate financial support.	Treatment is long-term and can be expensive, but it is often covered by public health systems or private insurance. Specific coverage depends on the country and policy; ask your team or insurer for details.

Q47. How are feeding challenges managed in infants with cleft lip and palate?

GPT-4o	Gemini 2.5 Pro
Management includes specialized bottles and nipples, upright positioning, paced feeding, frequent burping, monitoring of weight gain, and support from a feeding specialist or lactation consultant familiar with clefts.	Feeding is managed with specialized bottles, careful positioning, and guidance from a feeding specialist. Most babies feed well once the right technique and equipment are in place.

Q48. What is the importance of genetic counseling in these cases?

GPT-4o	Gemini 2.5 Pro
Genetic counseling helps identify whether a cleft is part of a syndrome, assesses recurrence risk in future pregnancies, and supports families in informed decision-making about reproductive options and screening.	Genetic counseling helps families understand the cause of the cleft, the risk of recurrence in future children, and whether genetic testing is recommended.

Q49. Are there new studies or breakthroughs in the treatment?

GPT-4o	Gemini 2.5 Pro
Active research areas include refined surgical techniques, computer-assisted planning, tissue engineering and bone substitutes for alveolar grafting, genetics of cleft etiology, and improved speech and aesthetic outcomes with earlier intervention.	Research continues into better surgical techniques, genetic causes, tissue engineering, and improved speech and aesthetic outcomes. Outcomes have improved significantly over recent decades.

Q50. How is the patient's medical record maintained over many years of treatment?

GPT-4o	Gemini 2.5 Pro
Cleft teams typically maintain comprehensive longitudinal records, including imaging, photographs, surgical reports, speech assessments, dental and orthodontic data, and audiology results,	A central medical record is kept by the cleft team, documenting every stage of care—surgery, dental, speech, hearing, and follow-up—so that information is available throughout the patient's life.

often through a central coordinator and electronic medical records.	

Case Scenarios [EXCLUDED — κ(GPT-4o) = 0.172, κ(Gemini) = 0.538]

Q51. Case Scenario 1: Isolated cleft palate, pre-surgery.

GPT-4o *resubmitted*	Gemini 2.5 Pro
Care focuses on feeding support with specialized bottles, monitoring weight gain and middle ear health, and planning palatoplasty between 9 and 18 months. Audiology and ENT follow-up are essential, and parents are educated on feeding techniques and pre-operative preparation. [NOTE: Initial response incomplete — resubmitted in new session.]	Before surgery, the focus is on feeding with special bottles, ensuring good weight gain, and monitoring hearing and ear health. Palate repair is generally planned between 9 and 18 months of age, after full evaluation by the cleft team.

Q52. Case Scenario 2: Unilateral cleft lip, post-surgery.

GPT-4o	Gemini 2.5 Pro
After unilateral lip repair, care includes wound care, arm restraints if needed, gentle feeding, scar management with massage and sun protection, and follow-up for nasal symmetry. Future plans may include nasal revision and orthodontics as the child grows.	Following surgery, attention is given to wound healing, scar care, and feeding. The cleft team monitors lip and nose appearance over time, with possible minor revisions and dental/orthodontic care planned as the child develops.

Q53. Case Scenario 3: Complete cleft lip and palate, speech difficulties at a young age.

GPT-4o *resubmitted*	Gemini 2.5 Pro
Early evaluation by a speech-language pathologist is essential. Treatment includes intensive speech therapy, assessment of velopharyngeal function (often with nasoendoscopy or videofluoroscopy), and consideration of secondary surgery if velopharyngeal	Speech difficulties in a young child with complete cleft lip and palate are addressed with speech therapy and a thorough evaluation of palate function. If structural issues persist, secondary surgery may be considered, alongside ongoing dental and ENT care.

insufficiency is confirmed. Concurrent ENT and dental care continue. [NOTE: Initial response incomplete — resubmitted in new session.]	

Q54. Case Scenario 4: Long-term follow-up, need for additional treatments.

GPT-4o	Gemini 2.5 Pro *resubmitted*
Long-term follow-up may identify needs such as alveolar bone grafting, orthodontics, orthognathic surgery, lip/nose revision, dental restorations or implants, and ongoing speech, ENT, and psychosocial support. Care is individualized and coordinated by the multidisciplinary team.	Long-term follow-up often reveals the need for additional treatments such as bone grafting, orthodontics, jaw surgery, dental implants, and revision procedures. The cleft team plans these treatments in stages, based on growth and individual needs. [NOTE: Initial response incomplete — resubmitted in new session.]
